# Supplementary material for: Movement of Chronic Wasting Disease Prions in Prairie, Boreal and Alpine Soils
Source: Pathogens. 2023 Feb 7;12(2):269. doi: 10.3390/pathogens12020269 (PMC9965917; doi:10.3390/pathogens12020269)
Supplement: Supplementary file 1 [file pathogens-12-00269-s001.zip › pathogens-2186329-supplementary.pdf]

SUPPLEMENTARY INFORMATION for paper

Movement of chronic wasting disease prions in prairie, boreal and alpine soils

Alsu Kuznetsova[1,2], Debbie McKenzie[2,3], Bjørnar Ytrehus[5,6], Kjersti Selstad Utaaker[5,7] and Judd M. Aiken[2,4]*

[1] Department of Renewable Resources, University of Alberta, Edmonton, AB T6G 2G8, Canada
[2] Centre for Prions and Protein Folding Diseases, University of Alberta, Edmonton, AB T6G 2M8, Canada
[3] Department of Biological Sciences, University of Alberta, Edmonton, Alberta, Canada
[4] Department of Agricultural, Food and Nutritional Sciences, University of Alberta, Edmonton, Alberta, Canada
[5] Norwegian Institute for Nature Research (NINA), Trondheim, Norway
[6] Department of Biomedicine and Veterinary Public Health Sciences, Swedish University of Agricultural Sciences, Uppsala, Sweden
[7] Faculty of Biosciences and Aquaculture, Nord University, Bodø, Norway
* Correspondence: to: Judd Aiken, e-mail: judd.aiken@ualberta.ca

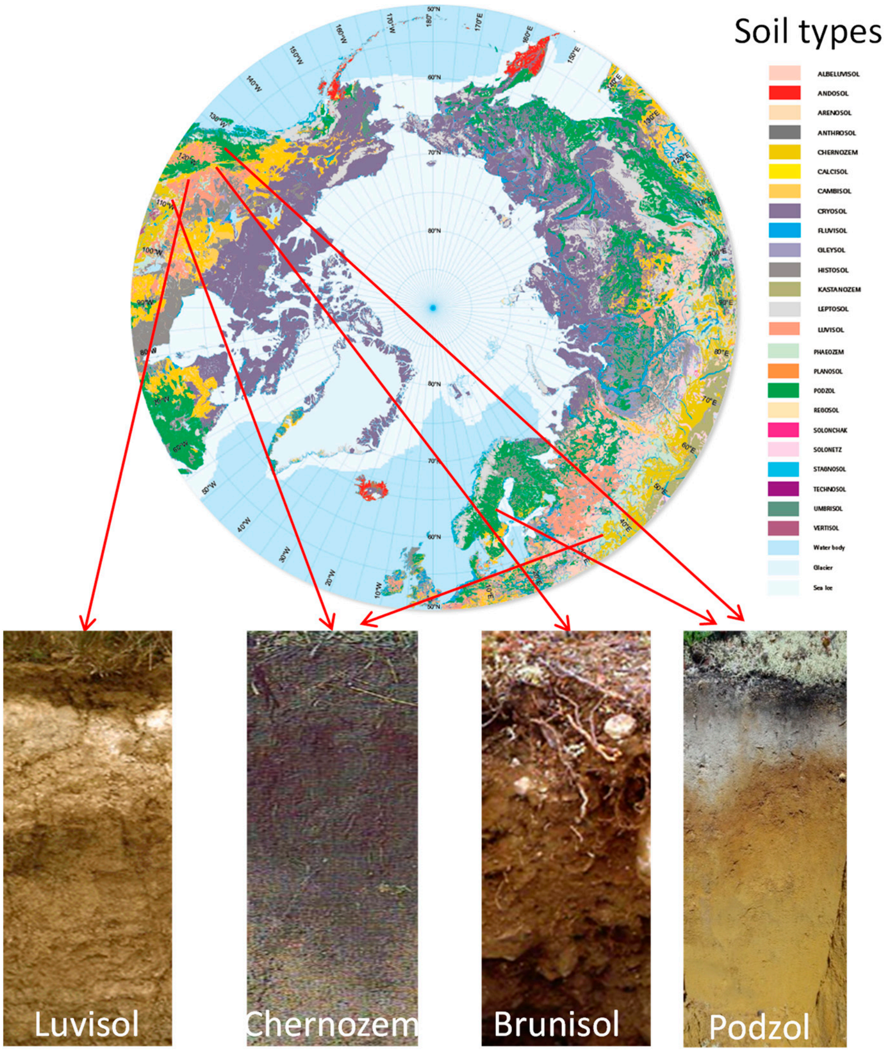

Figure S1. Soil map of circumpolar area with CWD-endemic region (Map source: https://esdac.jrc.ec.europa.eu/Library/Maps/Circumpolar/Documents/Circumpolar_atlas.pdf).

Experiment 1

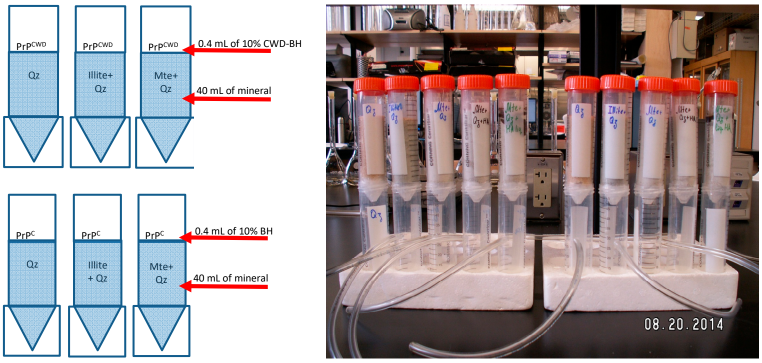

Experiment 2

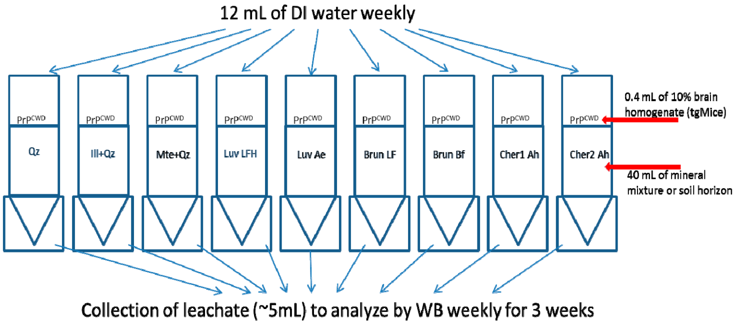

Figure S2. Experimental design of the PrP^{CWD} Transport Experiments. 24x100 mm tubes with perforated bottom were filled with 40 mL of minerals (quartz, Qz; illite, Ill; montmorillonite, Mte) or soil horizons (Luv – northern Luvisol, LFH and Ae horizons; Brun – mountain Brunisol, LFH and Bf horizon; Cher1 and Cher2 – central and southern Chernozems, Ah horizons). Ratios used - Mte:Qz 30:70 = sandy clay loam texture for columns incubated 6 weeks; Mte:Qz 50:50 = sandy loam texture for a column incubated 3 weeks. Brain homogenate (BH, 1% , tgMice) was applied on the surface. Columns were irrigated weekly with water, 12mL (simulating 90mm/month precipitation). Leachate was collected weekly to analyze presence of PrP.

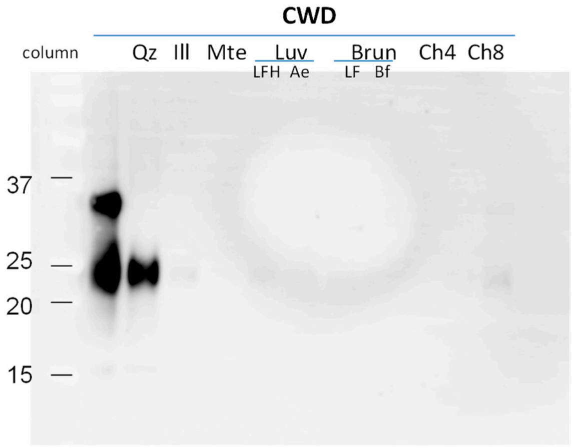

Figure S3. Leachate obtained at the third week from all 9 columns (column experiment 2): Qz – quartz, Ill – illite(50%)+quartz, Mte – montmorillonite (50%)+quartz , Luv – northern Luvisol, horizons LFH and Ae, Brun – mountain Brunisol, horizons LFH and Bf, Ch – Chernozems. ~ 5 mL of leachate was obtained from each column; 50uL of leachate were boiled at 100°C with SB and analyzed by western blot using mab Bar224.

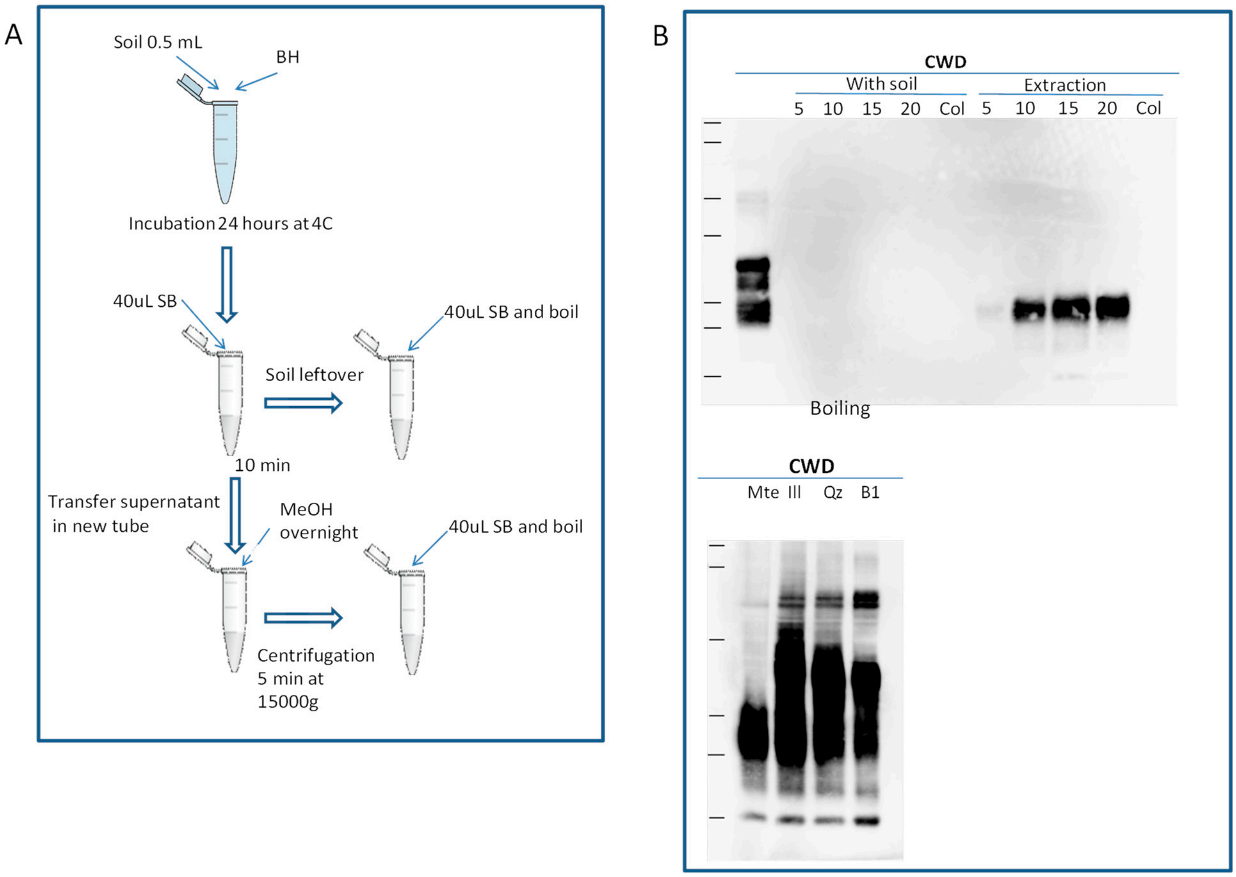

Figure S4. PrP extraction from solid phase. (A) Extraction protocol from soil. (B) Extraction 5 uL CWD from 0.5 mL of solid soil analyzed by western blot using Bar224 antibody.

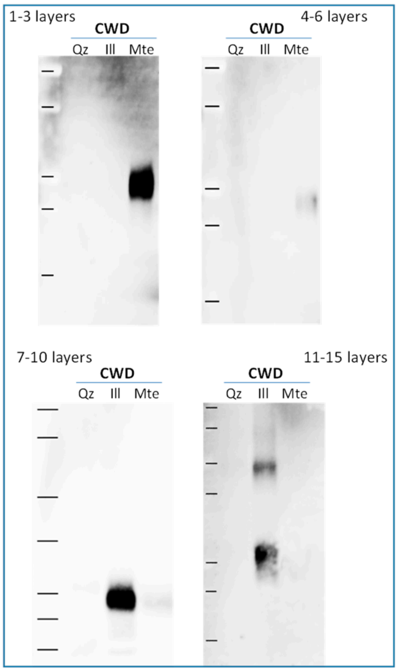

Figure S5. PrP signal extracted from solid phase of columns after 3 weeks incubation, mixed layers. Samples were boiled at 100°C with SDS and analyzed by western blot using Bar224 antibody.
